# Global User-Level Perception of COVID-19 Contact Tracing Applications: Data-Driven Approach Using Natural Language Processing

**DOI:** 10.2196/36238

**Published:** 2022-05-11

**Authors:** Kashif Ahmad, Firoj Alam, Junaid Qadir, Basheer Qolomany, Imran Khan, Talhat Khan, Muhammad Suleman, Naina Said, Syed Zohaib Hassan, Asma Gul, Mowafa Househ, Ala Al-Fuqaha

**Affiliations:** 1 Information and Computing Technology Division College of Science and Engineering Hamad Bin Khalifa University Doha Qatar; 2 Qatar Computing Research Institute Hamad Bin Khalifa University Doha Qatar; 3 Department of Computer Science and Engineering Faculty of Engineering Qatar University Doha Qatar; 4 Department of Cyber Systems University of Nebraska Kearney, NE United States; 5 Department of Computer Systems Engineering University of Engineering and Technology Peshawar Pakistan; 6 Department of Holistic Systems SimulaMet Oslo Norway; 7 Department of Statistics Shaheed Benazir Bhutto Women University Peshawar Pakistan

**Keywords:** COVID-19, sentiment analysis, contact tracing applications, NLP, text classification, BERT, fastText, transformers, RoBerta

## Abstract

**Background:**

Contact tracing has been globally adopted in the fight to control the infection rate of COVID-19. To this aim, several mobile apps have been developed. However, there are ever-growing concerns over the working mechanism and performance of these applications. The literature already provides some interesting exploratory studies on the community’s response to the applications by analyzing information from different sources, such as news and users’ reviews of the applications. However, to the best of our knowledge, there is no existing solution that automatically analyzes users’ reviews and extracts the evoked sentiments. We believe such solutions combined with a user-friendly interface can be used as a rapid surveillance tool to monitor how effective an application is and to make immediate changes without going through an intense participatory design method.

**Objective:**

In this paper, we aim to analyze the efficacy of AI and NLP techniques for automatically extracting and classifying the polarity of users’ sentiments by proposing a sentiment analysis framework to automatically analyze users’ reviews on COVID-19 contact tracing mobile apps. We also aim to provide a large-scale annotated benchmark data set to facilitate future research in the domain. As a proof of concept, we also developed a web application based on the proposed solutions, which is expected to help the community quickly analyze the potential of an application in the domain.

**Methods:**

We propose a pipeline starting from manual annotation via a crowd-sourcing study and concluding with the development and training of artificial intelligence (AI) models for automatic sentiment analysis of users’ reviews. In detail, we collected and annotated a large-scale data set of user reviews on COVID-19 contact tracing applications. We used both classical and deep learning methods for classification experiments.

**Results:**

We used 8 different methods on 3 different tasks, achieving up to an average F1 score of 94.8%, indicating the feasibility of the proposed solution. The crowd-sourcing activity resulted in a large-scale benchmark data set composed of 34,534 manually annotated reviews.

**Conclusions:**

The existing literature mostly relies on the manual or exploratory analysis of users’ reviews on applications, which is tedious and time-consuming. In existing studies, generally, data from fewer applications are analyzed. In this work, we showed that AI and natural language processing techniques provide good results for analyzing and classifying users’ sentiments’ polarity and that automatic sentiment analysis can help to analyze users’ responses more accurately and quickly. We also provided a large-scale benchmark data set. We believe the presented analysis, data set, and proposed solutions combined with a user-friendly interface can be used as a rapid surveillance tool to analyze and monitor mobile apps deployed in emergency situations leading to rapid changes in the applications without going through an intense participatory design method.

## Introduction

### Contract Tracing in the COVID-19 Response

Since the emergence of COVID-19, public authorities are trying their best globally to slow down the infection rate of the virus. As part of their efforts, several solutions, such as closing public places, imposing full or partial lockdowns, and limiting people’s contacts, have been implemented. Contact tracing has been globally recognized as one of the effective methods to slow down the infection rate of the virus [1]. To this aim, most of the initial efforts were based on manually tracing the contacts of infected persons. Manual contact tracing works only when the infected person knows who has been in physical contact with him or her, which reduces the effectiveness of the method. Moreover, manual contact tracing is a very time- and resource-consuming process [2,3].

The potential of contact tracing could be fully utilized if, ideally, the contact tracing mechanism can track the contact of an infected person on a very large scale. For instance, it would be ideally beneficial if authorities are able to track where the infected person has been and identify and notify the potential contacts of the patient. Technology, such as proximity sensors in smartphones and wearable devices, can help in such situations, allowing authorities to automatically notify potential cases more quickly and accurately [1,4]. To this aim, several mobile apps with a diversified set of features have been developed worldwide, each aligned with COVID-19–related policies, social values, and local infrastructure. However, the success of such applications is largely constrained by the number of users. According to Hinch et al [5], the potential of these mobile apps could be fully utilized if used by at least 80% of mobile users—which is 56% of the total population in the case of the United Kingdom, as reported by the authors, but generally depends on mobile penetration in the country.

To increase the number of users of these applications, different strategies and policies have been devised [6]. For instance, public authorities in several countries have made it mandatory for residents to install the contact tracing application to be able to access shopping malls, transportation, hospital, and other public places.

However, there are several concerns over these applications in terms of both effectiveness and privacy. For instance, since the applications require the tracking of individuals’ movements with GPS and other sensors to track their interactions, privacy concerns may arise [7,8]. Moreover, the literature has also identified a lack of understanding and unavailability of the technology (eg, smartphones) for a large portion of the population in limited-income countries as one of the main reasons for the lower effectiveness of such contact tracing applications [9].

### Motivation for This Study

The motivation for this study originated from the observation that, despite their successes, contact tracing applications have been subjected to public criticism and scrutiny globally due to concerns over privacy and other technical issues including enhanced battery consumption. We believe an analysis of users’ reviews on these applications will lead to a better understanding of the concerns over these applications. There are already some efforts in this regard [3,9,10]. However, the majority of the methods rely on exploratory and manual analysis of the users’ reviews, which is a resource- and time-consuming process. Moreover, some of the work also relies on existing general sentiment analysis platforms or tools, without training or fine-tuning the tools on COVID-19 application reviews. For instance, in [10], a commercial tool, AppBot, has been used for sentiment analysis of users’ reviews on only 9 mobile apps used in Europe. However, the tool relies on artificial intelligence (AI) models trained for generic sentiment analysis and returns 4 types of sentiment, namely positive, negative, neutral, and mixed. As a result, the outcome is not reliable, as the models are not trained on the task-specific data (ie, app reviews). For instance, a vast majority of the reviews are highlighting some technical issues, such as difficulties with registration, which also need to be analyzed. To address those limitations, we believe a task-specific model trained on a large-scale data set of manually annotated user reviews will help to make a better and context-specific classification of the reviews. Moreover, the existing literature relies on user reviews of fewer applications used in a specific region, which cover only a portion of the world’s population.

### Scope and Contributions

To facilitate an automatic sentiment analysis of users’ reviews on these applications, a large-scale manually annotated data set is needed to train and evaluate machine learning (ML) models for sentiment analysis. In this work, we collected and annotated a large collection of manually annotated user reviews of 46 different applications. More specifically, we collected and annotated a large-scale data set of Android and iOS mobile app users’ reviews for COVID-19 contact tracing.

We analyzed how AI models can help to automatically extract and classify the polarity of users’ sentiments and propose a sentiment analysis framework to automatically analyze users’ reviews on COVID-19 contact tracing mobile apps. Several algorithms were proposed and evaluated on the data set as a proof of concept to show the efficacy of automatic sentiment analysis of users’ reviews of these applications. After manually analyzing and annotating users’ reviews, we employed both classical (ie, multinomial Naïve Bayes [MNB], support vector machine [SVM], random forest [RF]) and deep learning (ie, neural networks [11], fastText [12], and different transformers [13]) methods for classification experiments. This resulted in 8 different classification models. Moreover, to the best of our knowledge, this is the first attempt to develop a large-scale benchmark data set for sentiment analysis of users’ reviews on COVID-19 contact tracing applications, which are from 46 distinct countries, from Google Play and Apple App Store. The proposed solutions combined with an interface can be used as a rapid surveillance tool to monitor how effective the app is and to make immediate changes without going through an intense participatory design method, which, although in normal circumstances is optimal, is not optimal in emergency situations in which a mobile device needs to be deployed immediately for the greater public good and with little-to-no user input from the beginning.

The main contributions of the work can be summarized as follows: We provide 34,534 manually labeled reviews based on the analysis of 40,000 reviews from 46 different COVID-19 contact tracing applications. The labels consist of sentiment polarities (ie, positive, neutral, and negative) and a label (technical issue). We provide an in-depth analysis of the data set that demonstrates different characteristics and insights. We share the data set and data splits with the research community for both reproducibility and further enhancements. We report benchmark results using 8 different classification experiments, which can serve as a baseline for future studies. We also propose a web application employing the proposed NLP techniques and with a user-friendly interface allowing stakeholders to quickly analyze people’s perceptions about such mobile apps.

### Related Work

To fight the COVID-19 pandemic, almost all research communities, such as health, NLP, and computer vision, have been playing a significant role. As a result, several interesting solutions aimed at different aspects of the pandemic have been proposed over the last year [14]. For instance, there have been efforts for early COVID-19 outbreak detection to help in emergency response preparedness [15]. Similarly, a large portion of the efforts aimed to address automatic diagnosis, prognosis, and treatment [15,16]. Fake news detection, risk assessment, logistic planning, and understanding of social interventions, such as monitoring social distancing, are the other key aspects of the pandemic that received the attention of the community [14,17,18]. Contact tracing is also one of the aspects of the pandemic that has been widely explored in the literature. For instance, the study by Lash et al [19] analyzed the mechanism and results of contact tracing in 2 different countries. The authors reported that an accurate and efficient mechanism of contact tracing can significantly reduce the infection rate of the virus. However, several challenges are associated with timely and accurate contact tracing of a COVID-19 patient. In this regard, a joint effort from the community and the use of more advanced methods relying on different technologies, such as GPS, Wi-Fi, Bluetooth, social graph, network-based APIs, and mobile tracking data, will help to a great extent [20,21]. Handheld devices, such as mobile phones, that are already embedded with such technologies are ideal platforms for deploying contact tracing solutions. Being a feasible solution, several mobile apps have already been developed in different parts of the world. In addition to basic contact tracing capabilities, different features are also implemented based on the domestic COVID-19 policies [22]. For instance, in different countries, such as Qatar and Australia, the applications are used to access different facilities. Similarly, in Saudi Arabia, an application is used to seek permission for going out during lockdown. In Table 1, we provide a list of some of the prominent contact tracing applications used in different parts of the world.

**Table 1:** COVID-19 contact tracing mobile apps used in this study.

S. No.	Country	Application	Technology
1	Australia	COVIDSafe	Bluetooth, Google/Apple
2	Austria	Stopp Corona	Bluetooth, Google/Apple
3	Bahrain	BeAware	Bluetooth, location
4	Bangladesh	Corona Tracer BD	Bluetooth, Google
5	Belgium	Coronalert	Bluetooth, Google/Apple
6	Bulgaria	ViruSafe	Location, Bluetooth, Google/Apple
7	Canada	COVID Alert	Bluetooth, Google/Apple
8	Cyprus	CovTracer	Location, GPS
9	Czech Republic	eRouska	Bluetooth, Google/Apple
10	Denmark	Smittestop	Bluetooth, Google/Apple
11	Estonia	HOIA	Bluetooth, DP-3T, Google/Apple
12	Fiji	CareFiji	Bluetooth, Google/Apple
13	Finland	Koronavilkku	Bluetooth, DP-3T
14	France	TousAntiCovid	Bluetooth, Google/Apple
15	Germany	Corona-Warn-App	Bluetooth, Google/Apple
16	Ghana	GH COVID-19 Tracker	Location, Google/Apple
17	Gibraltar	Beat Covid Gibraltar	Bluetooth, Google/Apple
18	Hungary	VirusRadar	Bluetooth, Google
19	Iceland	Rakning C-19	Location, Google/Apple
20	India	Aarogya Setu	Bluetooth, location, Google/Apple
21	Indonesia	PeduliLindungi	Bluetooth, Google/Apple
22	Ireland	Covid Tracker	Bluetooth, Google/Apple
23	Israel	HaMagen	Location, Google/Apple
24	Italy	Immuni	Bluetooth, Google/Apple
25	Japan	COCOA	Google/Apple
26	Kingdom of Saudi Arabia	Tawakkalna	Bluetooth, Google
27	Kingdom of Saudi Arabia	Tabaud	Google
28	Kuwait	Shlonik	Location, Google/Apple
29	Malaysia	MyTrace	Bluetooth, Google/Apple
30	Mexico	CovidRadar	Bluetooth
31	New Zealand	NZ COVID Tracer	QR codes, Google/Apple
32	North Macedonia	StopKorona	Bluetooth
33	Northern Ireland	StopCOVID NI	Bluetooth, Google/Apple
34	Norway	Smittestopp	Bluetooth, location, Google
35	Pakistan	COVID-Gov-PK	Bluetooth, GPS, Google/Apple
36	Philippines	StaySafe	Bluetooth, Google/Apple
37	Poland	ProteGO Safe	Bluetooth, Google
38	Qatar	Ehteraz	Bluetooth, location, Google/Apple
39	Singapore	TraceTogether	Bluetooth, Google/Apple
40	South Africa	COVID Alert SA	Bluetooth, Google/Apple
41	Switzerland	SwissCovid	Bluetooth, DP-3T, Google/Apple
42	Thailand	MorChana	Location, Bluetooth
43	Tunisia	E7mi	Google/Apple
44	Turkey	Hayat Eve Sıg˘ar	Bluetooth, location, Google/Apple
45	United Arab Emirates	TraceCovid	Bluetooth
46	United Kingdom	NHS COVID-19 App	Bluetooth, Google/Apple

Despite being a feasible solution for slowing down the infection rate, these applications are subject to criticism due to risks associated with them. In the literature, several issues, such as privacy, power consumption, and annoying alerts, have been reported. For instance, Bengio et al [23] analyzed and reported privacy issues associated with COVID-19 contact tracing applications. In addition to some recommendations on how to ensure users’ privacy, the authors also proposed a decentralized design for contact tracing by optimizing the privacy and utility trade-offs. Reichert et al [24] also analyzed privacy concerns of applications and proposed a privacy-preserving contact tracing mechanism relying on the privacy-preserving protocol secure multi-party computation [25] to ensure individuals’ privacy. Power consumption is another key challenge with contact tracing applications.

The literature also describes several interesting studies in which the feasibility of such mobile apps is assessed by analyzing people’s response or feedback on these applications [22,26,27]. For instance, in [28], an online survey was conducted to analyze citizens’ responses to HSE3, a contact tracing application used in Ireland. During the survey, a reasonable percentage of the participants showed their intention to use the application. However, the survey mainly aimed to analyze and identify different barriers to the use of such an application without analyzing the experience of the users with the application. To better analyze, understand, and evaluate users’ experiences and feedback on COVID-19 contact tracing applications, a detailed analysis of public reviews is required, which are available in Apple and Google Play Store. There are already some efforts in this direction. For instance, Rekanar et al [3] provided a detailed analysis of users’ feedback on HSE3 in terms of usability, functional effectiveness, and performance. However, the authors relied on manual analysis only, which is a time-consuming process. Another relevant work is reported in [10], in which an exploratory analysis of users’ feedback on 9 COVID-19 contact tracing applications used in Europe is provided. To this aim, the authors relied on a commercial app-review analytics tool, Appbot, to extract and mine the users’ reviews on the applications. To the best of our knowledge, the literature still lacks a benchmark data set to train and evaluate ML models for automatic analysis of users’ feedback on COVID-19 contact tracing applications. Moreover, the existing literature relies on user reviews of few applications used in a specific region, which covers only a portion of the population of the world. Hence, our work differs in a the following ways: We (1) analyzed reviews of a large number of applications used in different parts of the world, (2) manually annotated a data set and provided it for the community, and (3) provide detailed experimental results.

## Methods

### Overview

**Figure 1:**
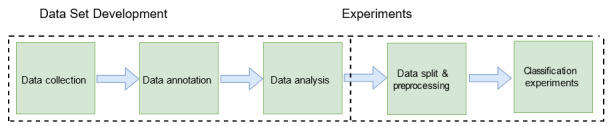
Block diagram of the proposed pipeline for sentiment analysis of users’ feedback on COVID-19 contact tracing mobile apps, roughly divided into 2 components, namely (1) data set development and (2) experiments.

In this section, we provide an overview of the methodology adopted in this paper. The complete pipeline of the proposed work is depicted in Figure 1. The work was mainly carried out in 2 different phases, including the (1) data set development phase and (2) experimental phase. In the development phase, as a first step, we scraped Google Play and App Store to obtain users’ reviews on COVID-19 contact tracing applications used in different parts of the world. After obtaining user reviews, a crowd-sourcing study was conducted to annotate the reviews for the training and evaluation of ML models for automatic sentiment analysis of the reviews. The annotation and other details obtained during the crowd-sourcing study were then analyzed, as detailed in later sections. In the experimental phase, before conducting experiments, data were preprocessed to make the data more meaningful for the AI models deployed in the experiments. During the experiments, we employed several AI models, as detailed in later sections. In the remainder of this section, we provide details of our data set development process, including our methodology for collecting, annotating, and analyzing the data set.

### Data Set Development

#### Data Collection

To obtain real-world user reviews for our analysis, we crawled reviews from 46 COVID-19 contact tracing applications used in the different parts of the world and hosted on Google Play and Apple’s App Store. These applications are listed in Table 1. The list of the applications to be covered in this work was obtained from online sources. During our search, we used different key words to make sure most of the applications were covered in the analysis. We note that, in this work, we considered reviews in the English language only when we made sure to analyze and annotate at least 50% of reviews of each application. However, to make sure the data set was balanced in terms of reviews from different applications, for some applications, such as Aarogya Setu, a lesser portion of the available reviews was analyzed. In addition to users’ reviews, we also obtained replies to the reviews, if any were available, as well as the ratings. However, for this study, we only used the reviews for the analysis and experiments. We note that the reviews were obtained from December 20, 2020, to December 25, 2020. It is important to mention that, in this work, we mainly aimed to collect a large collection of users’ reviews for training and evaluation of ML models to automatically analyze users’ reviews of the applications in the future. Thus, covering all reviews on a particular application was not our interest in this work. However, we tried our best to cover enough samples from all applications. Moreover, the main idea behind covering reviews from several applications instead of a single application was to train our models on a diversified data set, which will ultimately help our model to automatically analyze reviews on any application used in any part of the world.

#### Data Annotation

For the annotation of sentiment, typically 3 sentiment polarities are used: positive, negative, and neutral. From our initial analysis, we realized that applications could have technical problems; hence, we used another label, technical issues, for the annotation. Hence, our annotation consists of 4 labels: (1) positive, (2) negative, (3) neutral, and (4) technical issues. We note that the neutral reviews were the reviews that neither praised nor complained about the applications.

**Figure 2:**
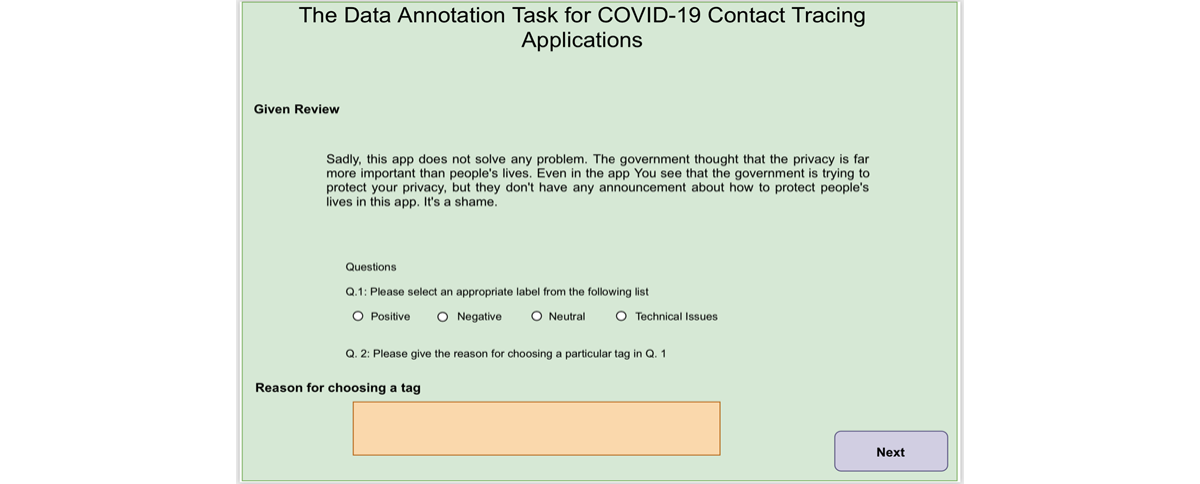
Screenshot of the annotation platform.

To facilitate the annotation process, we developed a web application through which user reviews on the applications were presented to the annotators to manually label them. In Figure 2, we present a screenshot of the annotation platform, which demonstrates the review and labels to be annotated (Q.1). In addition, we asked the annotators to briefly provide the reason behind their decision provided in response to Q.1. The question (Q.2) was used to evaluate the quality of the annotation (ie, whether the annotator carefully read the review). Moreover, we believe this question will provide useful information for the manual analysis of user feedback.

In total, 40,000 reviews were analyzed. To assure the quality of the annotations, each review was analyzed by at least 2 participants (graduate students from different age groups), and we considered the reviews and labels for which the majority of the annotators agreed. During the annotation process, we removed some reviews due to reasons such as (1) not being in English, (2) having a large number of emoticons or signs, and (3) irrelevancy (ie, not directly commenting on the applications). This process resulted in a total of 34,534 annotated reviews.

We made the data set publicly available for researchers to further explore the potential of NLP techniques for the automatic analysis of user feedback on contact tracing applications [29].

### Analysis

Overall, the data set covers a significant number of samples in each class. However, one of the classes, namely neutral, was composed of relatively fewer samples. In total, we had 15,587 reviews in the positive class, while the negative and technical issues classes were composed of 8178 and 9496 samples, respectively. The minority class (ie, neutral) contained a total of only 1271.

**Figure 3:**
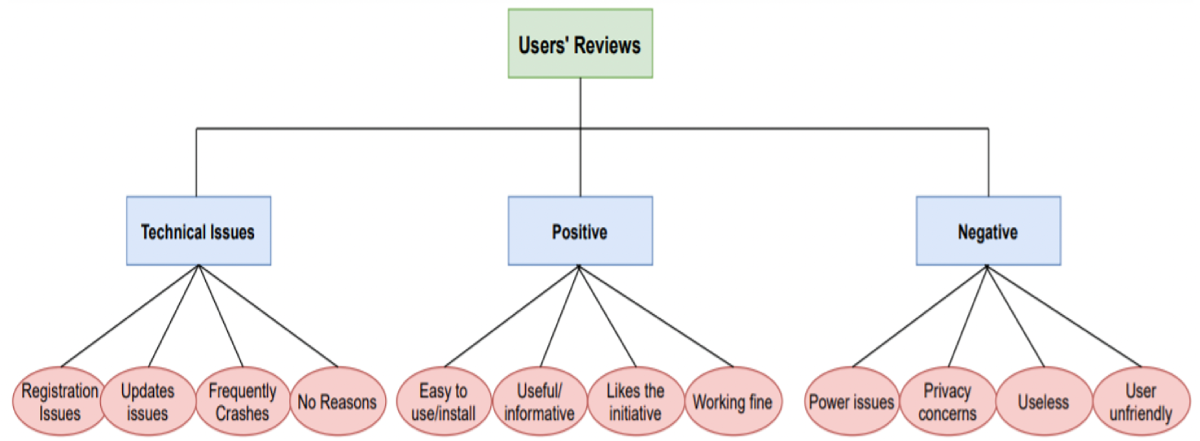
Taxonomy of main reasons or causes for the positive and negative reviews, as well as technical issues.

From the analysis of the second question (Q.2), we identified the reasons and information that influenced the participants’ decisions. In this section, we provide the statistics related to the second question (Q.2). In Figure 3, we provide a taxonomy of the most frequent reasons and causes for positive and negative reviews, along with the common technical issues.

In Table 2, we provide the distribution of the most common reasons and causes associated with the positive reviews, negative reviews, and technical issues, respectively.

**Table 2:** Common reasons for the feedback provided in the reviews (n=34,534).

Type of feedback		Frequency of responses, n (%)
**Positive feedback (n=15,587)**		
	Easy to install or use	1137 (7.3)
	Useful, informative, and helpful	5673 (36.4)
	Likes the idea or initiative	904 (5.8)
	Working fine	3226 (20.7)
	No reason or other	4645 (29.8)
**Technical issues (n=9496)**		
	Registration issues	4111 (43.3)
	Update issues	978 (10.3)
	Frequent crashes	1443 (15.2)
	No reason or other	2954 (31.1)
**Negative feedback (n=8178)**		
	Power consumption	1733 (21.2)
	Privacy concerns	1063 (13.0)
	Useless	2020 (24.7)
	Not user-friendly	1022 (12.5)
	No reason or other	2339 (28.6)

In the majority of positive reviews, users found the applications useful, informative, and helpful in the battle against COVID-19. Some sample positive reviews were:

Thank you very much. it’s very helpful and informative. it helps keep people away from suspicious areas.

A very good app for tracing and stopping coronavirus

Always getting updated information about the virus

Very useful and informative app.

A significant portion of positive reviews was also based on ease of installation, while some reviews mentioned that the application they were using is working fine, without further details. However, the most encouraging aspect was the fact that a significant percentage of users appreciated the idea, concept, and efforts made by the authorities for contact tracing to slow down the infection rate. Some sample reviews included:

A good initiative by the government

Good initiative to prevent the spread of corona virus, I appreciate who work behind this effort.

There was also a large number of short reviews in which the users simply showed their positive response without mentioning any particular reason. In addition to these, other common reasons for positive reviews highlighted by the users included some specific features of different applications in different parts of the world. For example, the Takkawalna app from the Saudi Government, which was used to seek permission to go out during lockdown, was praised for being a source of seeking permissions.

On the other hand, the key technical issues with these applications included registration and update issues. Moreover, a large number of reviews also highlighted that the application crashes or frequently stops working. In addition to these common issues, the reviews also hinted at certain technical issues, such as device compatibility and connectivity issues; lack of support for some languages, such as English; and not correcting QR codes from different applications. Some sample reviews highlighting technical issues in the applications included:

The app continually crashes.

I have business visa i am unable to register please give a solution on this.

I installed but i cant register yet.

I can’t update?

Install the apps, but keep showing connection error. Even restart the phone, also the same.

The most common issues included power consumption, uselessness, and privacy (Table 2). A significant number of reviews also highlighted that the majority of the applications are not user-friendly. There were also reviews depicting other issues, such as annoying notifications, unnecessary access to the gallery, slow response from the helpline, and unavailability of some key features, which could further improve the effectiveness of the applications. Some sample negative reviews included:

Too much personal information collected. Privacy risk. Non-compliant to international standards.

Allow too many permissions please ban this application. A total waste.

I have concerns with their data privacy.

This app is a battery hog.

**Figure 4:**
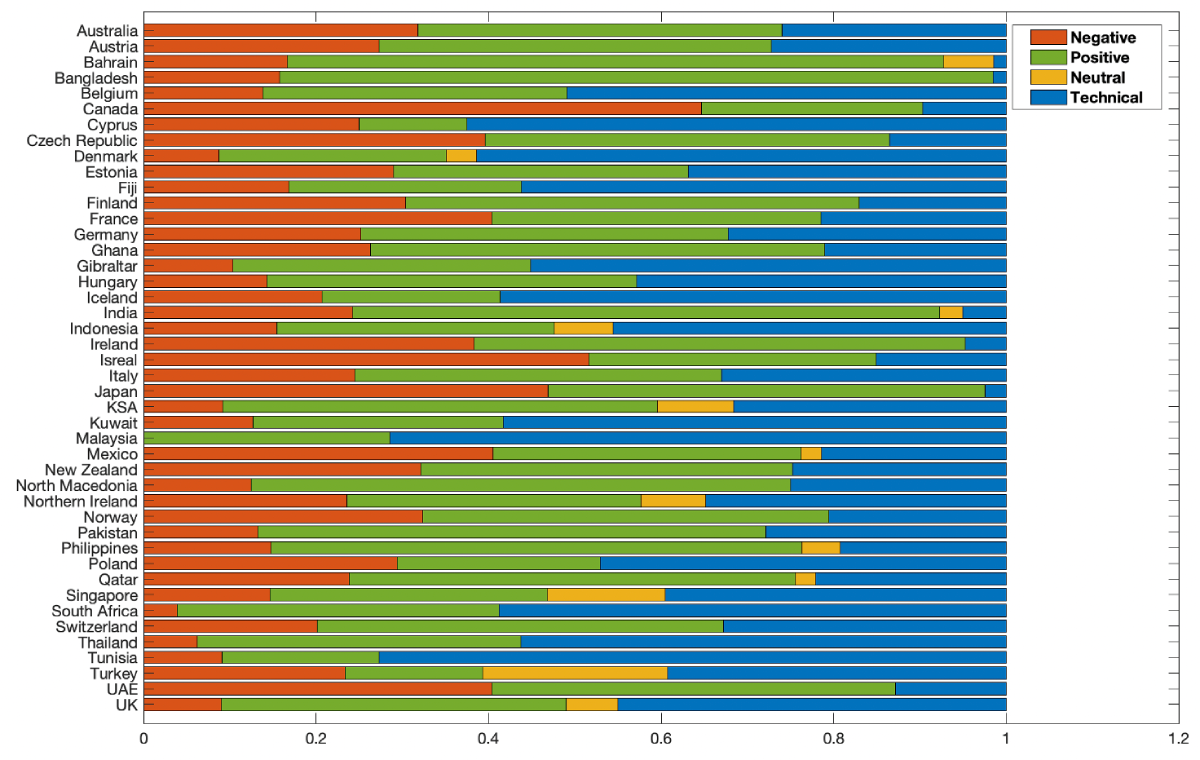
Distribution of negative, positive, and neutral reviews as well as technical issues reported for the applications in our data set, by country.

We also provide statistics by country in Figure 4, where we summarize the number and percentage of samples and reviews on the applications used in different countries belonging to each category. An important observation from the figure is the variation in the distribution of number of negative, positive, neutral, and reviews highlighting technical issues in different parts of the world. The variations in the number of reviews in each class depict how different responses to the use of the applications have been observed in different parts of the world. As can be seen, in certain countries, such as Japan, Israel, Canada, and Ireland, the ratio of negative reviews is high. The ratio of positive reviews is sufficient in most countries, which shows the trust of users in the applications. On the other hand, as expected, fewer neutral reviews from the majority of the countries were obtained for the data set. The data set also covers a significant ratio of the technical issues class in the majority of the countries. For instance, the ratio of the reviews highlighting technical problems in the applications is significantly high in Denmark, Tunisia, and Cyprus.

**Figure 5:**
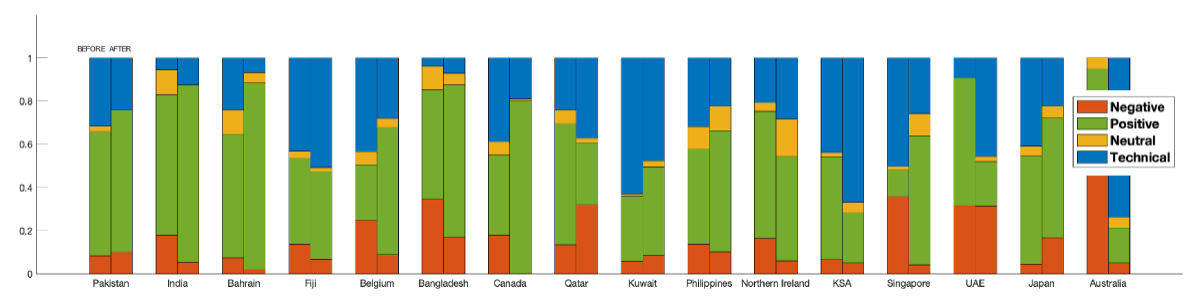
Preliminary temporal analysis reflecting the changes in the distribution of the sentiment classes over time. The data were compiled by analyzing the top 200 more recent (ie, December 25, 2020) and the initial 200 reviews from some of the applications that had a sufficient number of reviews.

To analyze the changes in the polarity of user sentiment over time, in Figure 5, we provide a preliminary temporal analysis to analyze the variation in the distribution of negative, positive, and neutral as well as technical issues over time. We note that, in this work, we provide a preliminary temporal analysis, which will be explored further in the future, and to this aim, we manually analyzed the 200 most recent reviews and the initial 200 reviews on applications having a reasonable time duration in the initial and more recent reviews. As seen in Figure 5, a higher overall variation is observed in the positive, negative, and neutral categories. As far as the individual applications are concerned, higher variation in the polarity of sentiments is observed for the applications used in Australia, Singapore, the United Arab Emirates, and Canada.

During the data analysis, though there were some doubts about privacy, we observed that, at the beginning, the initiative or idea of contact tracing was largely appreciated by the users in different parts of the world. Moreover, we also observed that the users of these applications faced device compatibility and registration issues with the application with time. Interestingly, in the case of most of the applications, the number of negative reviews increased with time. One of the possible reasons for the increase is the applications’ failure to achieve what they promised.

### Lexical Analysis

To understand the lexical content, we conducted an analysis of the number of tokens for each review. It can help to understand the characteristics of the data set. For example, for convolutional neural network and long short-term memory–based architectures, it is necessary to define the maximum sequence length. The minimum, maximum, and average numbers of tokens in the data set were 3, 198, and 18, respectively. Table 3 provides the statistics of the data set in terms of length of the different reviews in the data set.

We also analyzed the lexical content in each category to understand whether they are distinctive in terms of the lexical content top n-grams. This analysis also demonstrates the quality of the labeled data. We compared the vocabularies of all categories using the valence score [30,31], ϑ for every token, x, using the following equation:



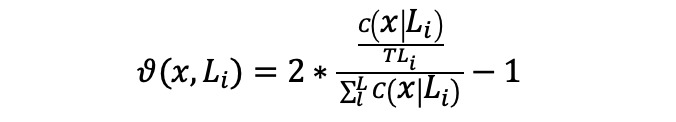



where C(.) is the frequency of the token x for a given class *L_i_.TL_i_* is the total number of tokens present in the class. In ϑ(x)ϵ[+1 –1], the value +1 indicates that the use of the token is significantly higher in the target class than the other classes. In Table 4, we present the most frequent bi- and trigrams with ϑ=1.0 for each category. From the table, we observe these n-grams clearly represent the class-wise information of the data.

**Table 3:** Number of reviews of different lengths in the overall data set.

Number of tokens	Reviews, n
0-20	23,602
21-40	6611
41-60	2463
61-80	1068
81-100	664
101-120	68
>120	60

**Table 4:** The most frequent class-wise n-grams based on valance scores.

Ranking	Negative	Positive	Technical issues
1	Battery went down	Best app	Error requesting
2	to delete this	Excellent app,	Cannot register.
3	overheating and battery	and helpful	what’s wrong
4	Not happy	Very nice and	fix this,
5	uninstall due to	feel safer	I can’t seem
6	Drains battery and	very good apps	Unable to proceed
7	massive drain	Good information	error while
8	too much battery,	save lives and	have error
9	Massive battery drain	very useful for	phone number. Tried

### Experiments

#### Task Description

As discussed earlier, we obtained a large number of samples for positive, negative, and technical issues (PNT), while fewer samples were obtained in the neutral class. Moreover, the reviews highlighting technical problems in the applications could also be treated as negative reviews. Thus, to cover different aspects of the problem, we divided it into 3 different tasks.

Task 1 involved ternary classification of PNT. We treated the problem as a ternary classification problem, where PNT were considered. The models trained for this task were expected to help identify the reviews highlighting technical problems in the applications along with the positive and negative reviews.

Task 2 involved binary classification (positive or negative [PN]). The negative and technical issues classes were merged into a single negative class to form 2 classes for a binary classification problem along with positive reviews (ie, PN). One of the main reasons for treating the task as a binary classification issue was the availability of fewer samples in the neutral class.

Task 3 involved ternary classification of 3 classes: positive, negative, or neutral (PNN). We note that, in this task, the negative class is the combination of the original negative and technical issues classes.

All these tasks helped analyze how the performance of the proposed sentiment analyzer varies with different sets of annotations.

#### Data Splits

For the classification experiments, we divided the data set into training, validation, and test sets, at proportions of 60.3%, 6.7%, and 30.0%, respectively. While dividing the data set, we used stratified sampling to maintain class distribution across different sets. The data split or distribution was performed for each task separately, which resulted in a different number of samples for the training, validation, and test sets for each task. The data split for each task will be made publicly available, separately, to ensure a fair comparison in future work. Tables 5-7 summarize the distribution of the data into the training, validation, and test sets used in Task 1, Task 2, and Task 3, respectively.

**Table 5:** Data split and distribution of class labels for Task 1.

Class	Train	Validation	Test	Total
Positive	9370	1041	5176	15,587
Negative	5000	556	2622	8178
Technical issues	5686	632	3178	9496
Total	20,056	2229	10,976	33,261

**Table 6:** Data split and distribution of class labels for Task 2.

Class	Train	Validation	Test	Total
Positive	9342	1038	5207	15,587
Negative	10,715	1191	5770	17,676
Total	20,057	2229	10,977	33,263

**Table 7:** Data split and distribution of class labels for Task 3.

Class	Train	Validation	Test	Total
Positive	9364	1040	5183	15,587
Negative	10,690	1188	5798	17,676
Neutral	770	85	416	1271
Total	20,824	2314	11,398	34,534

#### Data Preprocessing

Before proceeding with the experiments, the data were also cleaned by removing unnecessary tokens, such as non-ASCII characters, punctuation (replaced with whitespace), and other signs.

### Models

For this study, our classification experiments consisted of multiclass classification using both classical and deep learning algorithms as detailed in the following sections.

#### Classical Algorithms

For this study, we used several classical algorithms such as MNB [32], SVM [33], and RF [34]. As a feature representation with these algorithms, we used the bag-of-ngrams, which is one of the most commonly used methods for text classification and retrieval applications, applied with classical algorithms. This has been widely used as a simple, yet effective and computationally efficient, method. Motivated by its better performance in similar types of text classification applications, such as fake news and flood detection in Twitter text [17,35,36], we experimented with this representation using the mentioned classical algorithms.

#### fastText

fastText is an NLP library that aims to provide efficient word embedding and text classification at a faster speed compared with traditional deep learning solutions [12]. For word embedding, the model relies on the Continuous Bag of Words, which is based on a shallow neural network, strategy by predicting a word via its neighbors. To ensure training at a higher speed, the model relies on a hierarchical classification mechanism by replacing the traditional soft-max function with a hierarchical one resulting in a reduced number of parameters.

#### Transformers

Bidirectional Encoder Representations from Transformers (BERT) [13] is a state-of-the-art pretrained model that has demonstrated success in many downstream NLP tasks. It is typically used for downstream classification problems either by using embedding representations as features or fine-tuning the model. The main strength of the model comes from pretraining on a very large text data set that allows the model to understand and interpret text easily in different NLP applications. Moreover, the model also possesses the ability to learn from context. For this study, we used different transformer models, including BERT [13], RoBERTa [37], XLM-RoBERTa [37], and DistilBERT [38].

### Evaluation Metrics

To measure the performance of each classifier, we used weighted average precision (P), recall (R), and F1. We used weighted metrics as they have the capability to take into account the class imbalance distribution.

### Classification Experiments

To train the classifiers using MNB, SVM, and RF, we converted the text into bag-of-n-gram vectors weighted with logarithmic term frequencies (tf) multiplied with inverse document frequencies (idf). To utilize contextual information, such as the n-grams that are useful for classification, we extracted unigram, bigram, and trigram features.

We used grid-search to optimize the parameters for MNB, SVM, and RF. For MNB, we optimized Laplace smoothing the α parameter with 20 values between 0 and 1. For SVM, we optimized linear kernel with C parameters with 30 values ranging from 0.00001 to 10 and radial-basis-function kernel with C and γ parameters (for γ, we used 10 values from 1e-5 to 1e-1). For RF, we optimized the number of trees (10 values from 200 to 2000) and the depth of the tree (11 values from 10 to 110). Choosing such ranges of values depends on the available computational resources, as they are computationally expensive.

For fastText, we use pretrained embeddings trained on Common Crawl and default hyperparameter settings [39].

For transformer-based models, we use the Transformer Toolkit [40]. We fine-tuned each model using the hyperparameter settings in Table 8, with a task-specific layer on top of the model. As reported in [13], training with pretrained transformer models shows instability; hence, we performed 10 runs of each experiment using different random seeds and chose the model that performed the best on the development set. To train the transformer-based models for each task, we fine-tuned the model 10 epochs with “categorical cross-entropy” as the loss function and used the hyperparameter settings provided in Table 8.

The detailed number of parameters for each model, which demonstrates the size of the models, were as follows:

- BERT (bert-base-uncased): This model was trained on lower-case English text. It consists of 12 layers, 768 hidden states, 12 heads, and 110 million parameters.
- DistilBERT (distilbert-base-uncased): This is a distilled version of the BERT model consisting of 6 layers, 768 hidden states, 12 heads, and 66 million parameters.
- RoBERTa (roberta-large): RoBERTa, using the BERT-large architecture, consists of 24 layers, 1024 hidden states, 16 heads, and 355 million parameters.
- XML-RoBERTa (xlm-roberta-large): It consists of 355M parameters with 24 layers, 1027 hidden states, 4096 feed-forward hidden states, and 16 heads.

**Table 8:** Hyperparameter settings used during the experiments.

Parameters	Value
Batch size	8
Learning rate (Adam)	2e-5
Number of epochs	10
Max sequence length	128

## Results

### Task 1: Ternary Classification (PNT)

Table 9 provides the experimental results for Task 1 in terms of weighted accuracy, precision, recall, and F1 score. Overall better results were obtained with transformers compared with the classical and deep learning–based methods. One of the main reasons for the better performance of the transformers is their text interpretation capabilities. Though no significant differences were observed in the performances of the different transformers, a slight improvement was observed for RoBERTa over the rest of transformers.

To better analyze the performance of the proposed methods, we also provide the class-wise performance. Overall, reasonable results were obtained for all 3 classes; however, the performance of all the methods was higher for the positive class. One of the possible reasons for the comparatively lower performance for the other 2 classes is the lower interclass variation. As detailed earlier, reviews in the negative and technical issues classes contained similar types of words, and there were higher chances of confusion in the classes. The experimental results of Task 1 provide the basis for Task 2, where the negative and technical issues classes were merged.

**Table 9:** Experimental results for Task 1: ternary classification of positive, negative, and technical issues (PNT).

Method	Positive			Negative				Technical issues				Overall (weighted average)			
	P^a^	R^b^	F1	P	R	F1	P		R	F1	Acc^c^		P	R	F1
MNB^d^	.910	.892	.901	.679	.664	.671	.751		.789	.769	.808		.809	.808	.808
RF^e^	.854	.923	.887	.809	.538	.646	.729		.833	.777	.805		.806	.805	.797
SVM^f^	.946	.867	.905	.660	.707	.683	.745		.803	.773	.810		.820	.810	.814
fastText	.930	.904	.917	.713	.691	.702	.752		.806	.778	.825		.827	.825	.825
DistilBERT^g^	.943	.934	.939	.753	.714	.733	.778		.824	.800	.849		.850	.849	.849
BERT	.938	.936	.937	.750	.718	.734	.786		.817	.801	.850		.849	.850	.849
RoBERTa	.943	.946	.945	.754	.716	.734	.788		.817	.802	.854		.853	.854	.853
XML-RoBERTa	.941	.946	.943	.744	.705	.724	.783		.811	.797	.849		.848	.849	.848

^a^P: precision.

^b^R: recall.

^c^Acc: accuracy.

^d^MNB: multinomial Naïve Bayes.

^e^RF: random forest.

^f^SVM: support vector machine.

^g^BERT: Bidirectional Encoder Representations from Transformers.

### Task 2: Binary Classification (PN)

Table 10 provides experimental results for Task 2, where the models had to differentiate between positive and negative reviews. As expected, the performance was improved significantly on Task 2, which proves our hypothesis that negative and technical issues classes have similar content. Moreover, similar to Task 1, transformers outperformed the rest of the methods.

As seen in the table, in contrast to Task 1, no significant differences were observed in the performance of the methods on different classes, which indicates that reviews highlighting technical problems in the applications evoked negative emotions or sentiments. Moreover, no significant variation in the performance of the methods on a particular class was observed.

**Table 10:** Experimental results for Task 2: binary classification (positive or negative [PN]).

Method	Positive				Negative				Overall (weighted average)			
	P^a^	R^b^	F1	P		R	F1	Acc^c^		P	R	F1
MNB^d^	.925	.873	.898	.891		.936	.913	.906		.907	.906	.906
RF^e^	.902	.879	.891	.894		.914	.904	.898		.898	.898	.898
SVM^f^	.944	.876	.909	.895		.953	.923	.916		.918	.916	.916
fastText	.947	.890	.917	.905		.955	.929	.924		.925	.924	.924
DistilBERT^g^	.947	.932	.939	.939		.953	.946	.943		.943	.943	.943
BERT	.947	.936	.941	.943		.953	.948	.945		.945	.945	.945
RoBERTa	.948	.942	.945	.948		.953	.951	.948		.948	.948	.948
XML-RoBERTa	.953	.930	.942	.939		.959	.949	.945		.946	.945	.945

^a^P: precision.

^b^R: recall.

^c^Acc: accuracy.

^d^MNB: multinomial Naïve Bayes.

^e^RF: random forest.

^f^SVM: support vector machine.

^g^BERT: Bidirectional Encoder Representations from Transformers.

### Task 3: Ternary Classification (PNN)

Table 11 provides the experimental results for Task 3, where the models had to differentiate among positive, negative, and neutral reviews. Similar to sprevious 2 tasks, transformers produced better results compared with the classical and deep learning–based methods. As seen in the table, better results were reported for all the methods on positive and negative classes. However, the performance of the proposed methods was significantly lower, especially for the bag of words and ngram with the Naive Bayes classifier. One of the main reasons for the lower performance for the neutral class was the fewer samples in the class, as described earlier. We note that Task 2 and Task 3 were performed separately to analyze the impact of the fewer samples in the neutral class.

**Table 11:** Experimental results for Task 3: ternary classification (positive, negative, or neutral [PNN]).

Method	Positive				Negative				Neutral				Overall (weighted average)			
	P^a^	R^b^	F1	P		R	F1	P		R	F1	Acc^c^		P	R	F1
MNB^d^	.902	.873	.888	.854		.935	.892	.379		.027	.050	.874		.859	.874	.860
RF^e^	.875	.881	.878	.862		.916	.888	.333		.005	.010	.866		.844	.866	.851
SVM^f^	.926	.844	.883	.881		.914	.897	.211		.330	.257	.861		.877	.861	.868
fastText	.947	.890	.917	.905		.955	.929	.463		.177	.256	.891		.883	.891	.883
DistilBERT^g^	.932	.918	.925	.913		.934	.923	.364		.312	.336	.904		.901	.904	.902
BERT	.933	.927	.930	.913		.940	.926	.387		.261	.312	.909		.903	.909	.905
RoBERTa	.933	.931	.932	.919		.941	.930	.386		.269	.317	.912		.906	.912	.909
XML-RoBERTa	.941	.932	.936	.923		.936	.929	.341		.319	.333	.911		.910	.911	.911

^a^P: precision.

^b^R: recall.

^c^Acc: accuracy.

^d^MNB: multinomial Naïve Bayes.

^e^RF: random forest.

^f^SVM: support vector machine.

^g^BERT: Bidirectional Encoder Representations from Transformers.

**Figure 6:**
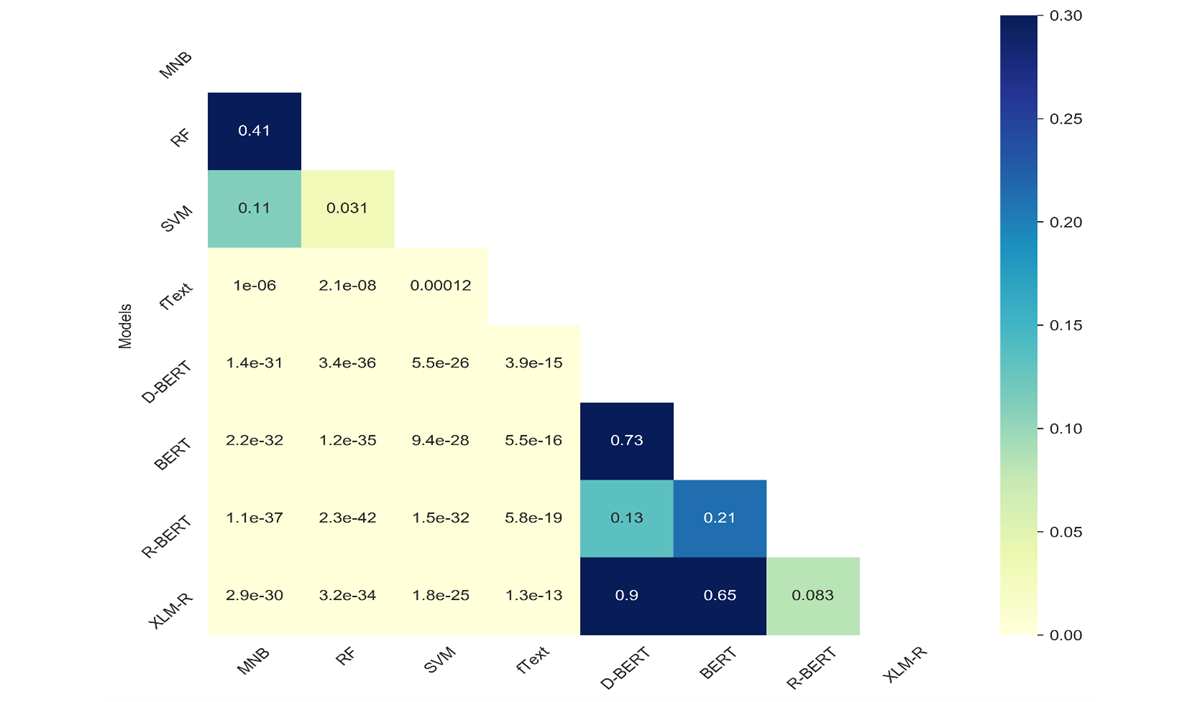
Results of the statistical significance (McNemar) test comparing the different methods for Task 1. BERT: Bidirectional Encoder Representations from Transformers; MNB: multinomial Naïve Bayes; RF: random forest; SVM: support vector machine.

**Figure 7:**
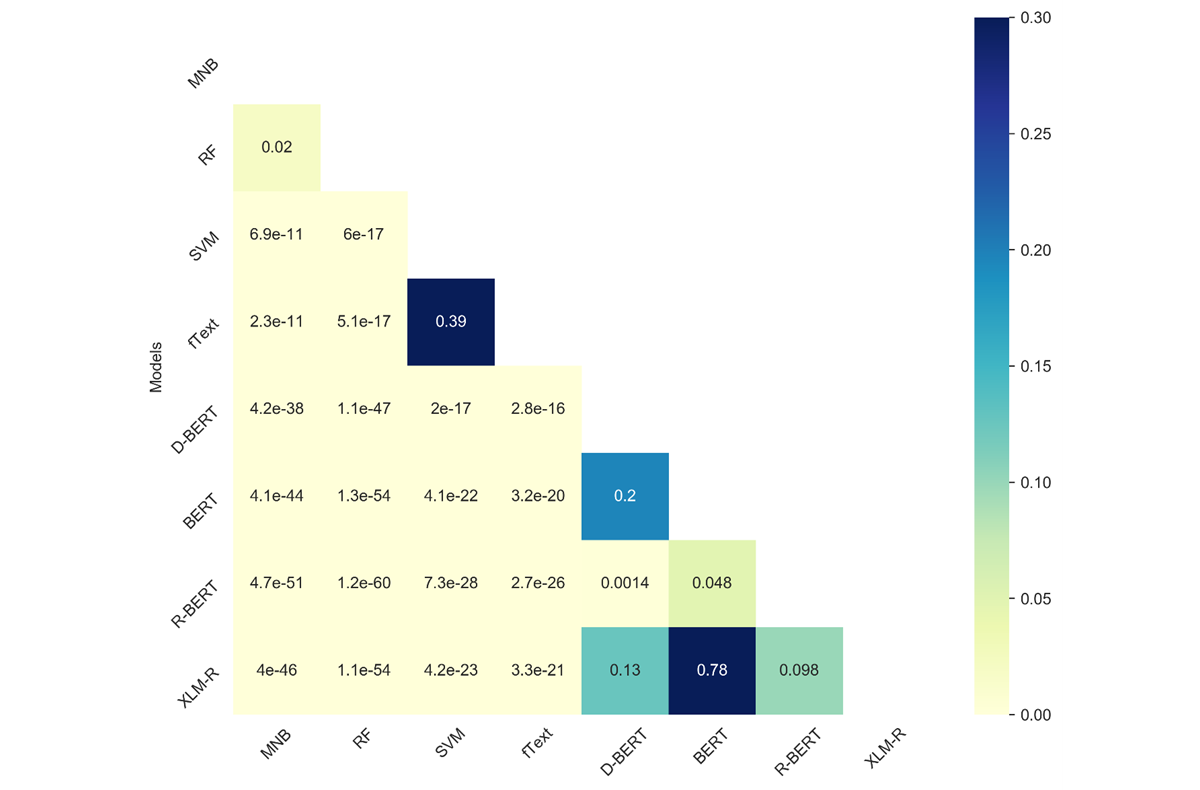
Results of the statistical significance (McNemar) test comparing the different methods for Task 2. BERT: Bidirectional Encoder Representations from Transformers; MNB: multinomial Naïve Bayes; RF: random forest; SVM: support vector machine.

**Figure 8:**
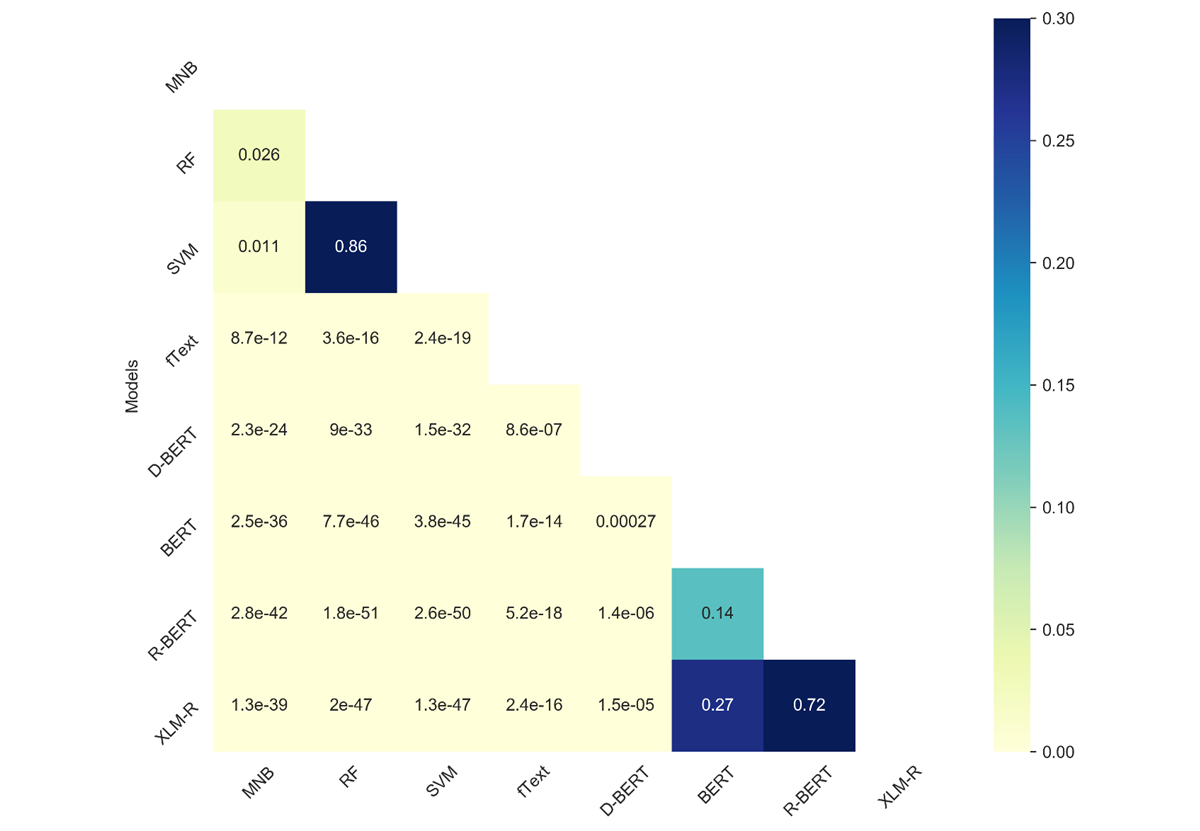
Results of the statistical significance (McNemar) test comparing the different methods for Task 3. BERT: Bidirectional Encoder Representations from Transformers; MNB: multinomial Naïve Bayes; RF: random forest; SVM: support vector machine.

We observed that the performance difference across different classical or transformer-based models was low; to understand whether such differences were statistically significant, we conducted statistical significance tests. We used the McNemar test for the binary classification task (ie, Task 2) and Bowker test for Tasks 1 and 3. More details of this test can be found in [41]. As can be seen in Figures 6-8, our findings suggest that, across all the tasks, the test results were statistically significantly different between transformers and the other models. The value in the cell represents the *P* value, and the light yellow color represents statistical significance (*P*<.05). Among the classical algorithms, the differences in the test results were not that significant. Similarly, among the transformer-based models, the results were low, which is also reflected in the overall F1 scores for the different tasks.

The variation in the performances of the models on the different tasks could be associated with the categories of reviews (ie, positive, negative, neutral, and technical issues) covered in each task. For instance, in the ternary classification Task 1, where 3 categories, namely positive, negative, and technical issues, were considered, the performance was lower due to the similarities between negative reviews and reviews highlighting technical issues. Similarly, in the binary classification Task 2, the performance was significantly improved when negative and technical issues classes were merged into a single class. On the other hand, in the ternary classification Task 3, the performance was degraded due to the lower number of samples in the neutral class.

### Dashboard of the Potential Rapid Analysis and Feedback Tool

**Figure 9:**
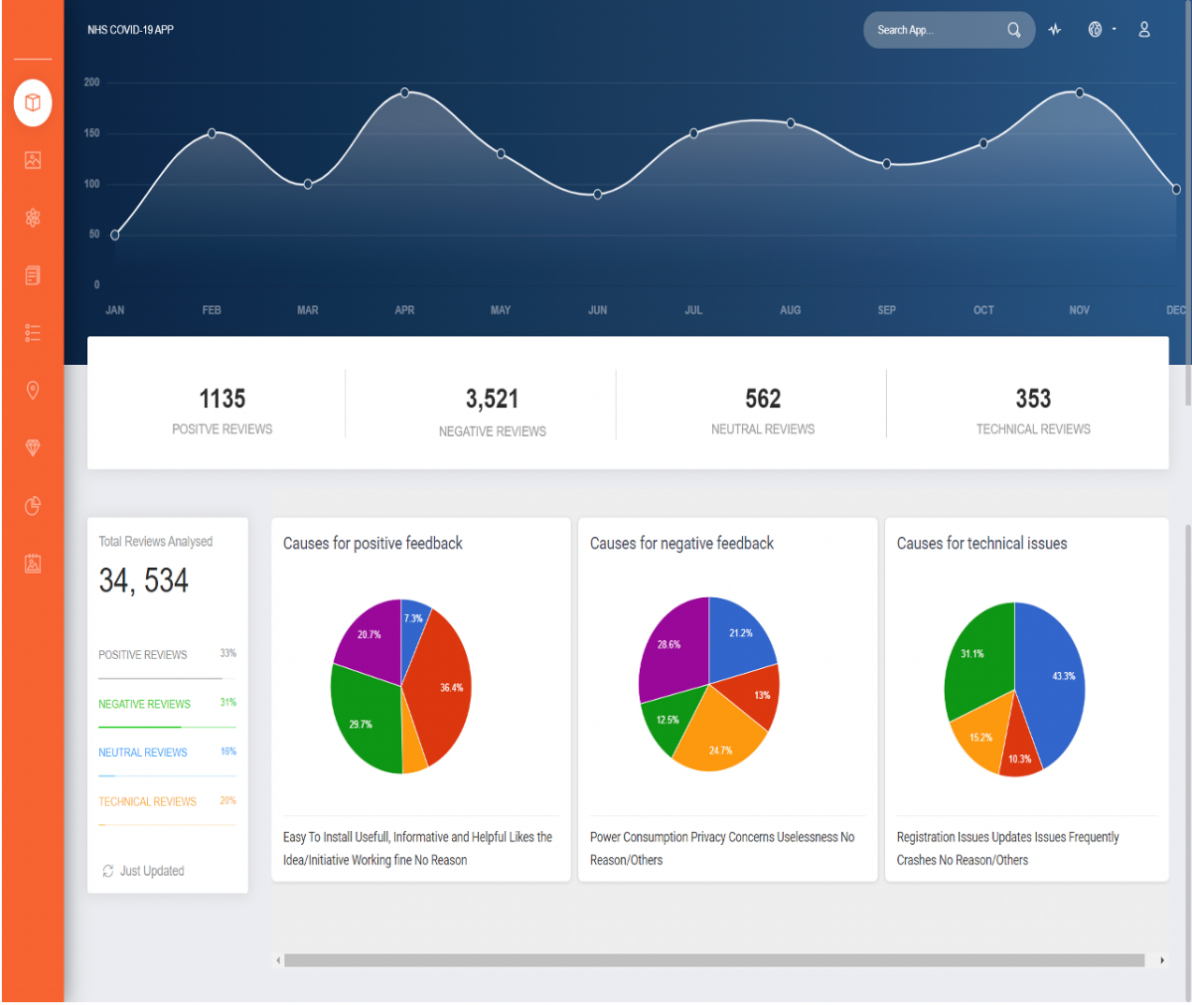
Screenshot of the potential tool based on the proposed solutions.

To facilitate different stakeholders (ie, the users of the proposed sentiment analyzer of users’ reviews of COVID-19 contact tracing applications), we also aimed to develop a web application with a user-friendly interface. Figure 9 provides a screenshot of the dashboard of the potential sentiment analyzer web application. In the current implementation, as seen in the figure, the web application provides the distribution of the positive, negative, neutral, and technical reviews along with a searching function to analyze user feedback on a specific application. It is important to mention that the proposed tool could be extended to other health care applications by training the models on relevant data sets.

## Discussion

### Principal Findings

Contact tracing of COVID-19 patients has been globally recognized as one of the most effective ways of controlling the infection rate. However, there are several limitations of the existing mechanisms. Manual contact tracing is a tedious and time-consuming process. Moreover, it is difficult to keep track of all potential contacts of a patient. Digital solutions, such as the use of mobile apps, have been considered as a promising solution, with which a patient’s contacts can be traced and informed quickly. However, there are several concerns over the working mechanism and performance of the applications. This work has revealed different facets of the COVID-19 contact tracing applications, advantages, drawbacks, and users’ concerns over these applications.

Our finding suggests that the idea or initiative for contact tracing via a mobile app is highly appreciated by people worldwide. In addition to contact tracing, the applications have also proved useful in implementing and ensuring public policies on COVID-19. However, there are also some concerns over the working mechanism and the effectiveness of the applications. In this regard, the analysis of users’ reviews of these applications helps to better understand and rectify the concerns over the applications.

We observed that the majority of the reviews lie within 3 categories, namely positive, negative, and technical issues. On the other hand, very few neutral reviews were observed. Privacy in terms of tracking via GPS and access to the gallery and other information by the applications were the main concerns. Moreover, a vast majority of the users of these applications in different parts of the world was not happy with the high power consumption of the applications. The majority of the users also faced some technical problems while using the applications. Some key technical issues included device compatibility, registration, slow updates, connectivity issues, and lack of support for some languages (eg, English). Another important observation is that the distribution of negative, positive, neutral, and technical issues may vary over time.

As far as the performance of the AI models is concerned, a better overall performance was observed for all the models in sentiment analysis of users’ reviews, allowing efficient analysis of users’ responses to the application more quickly. Among the models deployed for sentiment analysis in this work, the transformers were most effective. This shows the efficacy of AI and NLP techniques in automatic analysis of COVID-19 contact tracing applications.

### Conclusions

In this paper, we focused on the sentiment analysis of use reviews on COVID-19 contact tracing mobile apps and analyzed how users react to these applications. To this aim, a pipeline was composed of multiple phases, such as data collection; annotation via a crowd-sourcing activity; and development, training, and evaluation of AI models for the sentiment analysis. The existing literature mostly relies on manual or exploratory analysis of users’ reviews on the application, which is a tedious and time-consuming process. Moreover, in existing studies, generally, data from fewer applications were analyzed. In this work, we showed how automatic sentiment analysis can help analyze users’ responses to the applications more quickly. Moreover, we also provided a large-scale benchmark data set composed of 34,534 reviews from 46 different applications. We believe the presented analysis and data set will support future research on the topic.

We believe many interesting applications and analysis can be conducted keeping the data set as a baseline. Temporal and topical analyses are the key aspects to be analyzed in the future.

## Abbreviations

AI: artificial intelligence
BERT: Bidirectional Encoder Representations from Transformers
ML: machine learning
MNB: multinomial Naïve Bayes
PN: positive or negative
PNN: positive, negative, or neutral
PNT: positive, negative, and technical issues
RF: random forest
SVM: support vector machine
